# Analytical Analysis for a Uniformly Loaded Circular Plate of Functionally Graded Piezoelectric Materials Using Mian and Spencer Theory

**DOI:** 10.3390/ma19142942

**Published:** 2026-07-08

**Authors:** Huiduo Fan, Lulu Shen, Zaihu Zhu, Bo Yang

**Affiliations:** 1 Department of Civil Engineering, Zhejiang Sci-Tech University, Hangzhou 310018, China; 19857129053@163.com (H.F.); lulushen@zstu.edu.cn (L.S.); zhuzaihu1177@163.com (Z.Z.); 2 School of Construction Engineering, Zhejiang College of Construction, Hangzhou 311231, China

**Keywords:** functionally graded piezoelectric material, electro-elastic response, Mian and Spencer plate theory, elasticity solution, circular plate

## Abstract

Mian and Spencer developed a procedure for constructing exact solutions to the equations of linear elasticity in thick isotropic functionally graded plates. In this study, the Mian and Spencer plate theory is extended from elastic material to piezoelectric material by incorporating intrinsic electro-elastic coupling. An electric potential function is constructed by analogy with the displacement components, and the corresponding functions are obtained through stepwise integration using the mechanical and electrical boundary conditions on the top and bottom surfaces of the plates. Exact 3D elasticity solutions are then derived for a uniformly loaded FGPM circular plate under simply supported and clamped boundary conditions, with the material coefficients allowed to vary arbitrarily along the thickness direction z. The analytical solutions are validated through comparison with results reported in the literature. Finally, a systematic parametric study is performed to investigate the effects of the material gradient index, boundary conditions, thickness-to-radius ratio, and piezoelectric effect on the electro-elastic effect of FGPM circular plates.

## 1. Introduction

Since the concept of functionally graded materials (FGMs) was introduced into conventional multilayered hybrid piezoelectric structures [1,2], functionally graded piezoelectric material (FGPM) structures have attracted considerable interest in active and passive control applications for intelligent electromechanical devices because of their intrinsic electro-elastic coupling and gradation of material properties [3,4,5]. In FGPM structures, the material composition and properties vary continuously and smoothly along predesigned directions, thereby avoiding the internal interfaces and stress concentrations that are commonly induced by abrupt material-property mismatches in multilayered plates. Owing to these advantages, FGPMs have strong potential in advanced engineering applications, including high-performance sensors, micro-electromechanical systems, ultrasonic transducers, and precision actuators for aerospace and biomedical engineering. Accurate characterization of their three-dimensional (3D) electro-elastic behavior under coupled mechanical and electrical loading is therefore essential for reliable design and optimization. To support the broader use of FGPM in smart engineering applications, the three-dimensional (3D) electro-elastic responses of FGPM plates under external loads have been investigated using various numerical and semi-analytical methods. For instance, Ma et al. [6] employed isogeometric analysis combined with an improved moth-flame optimization algorithm for the static analysis of bi-directional FGMs with piezoelectric layers. Zhang et al. [7] analyzed nonlinear thermo-electro-mechanical responses of FGPM plates subjected to strong electric fields using the isogeometric analysis combined with the finite element method. Because full 3D numerical methods can be computationally expensive, semi-analytical methods have also been developed for the quasi-3D analysis of FGPM plates. For circular geometries, Sladek et al. [8] analyzed the bending of circular piezoelectric plates with functionally graded properties using the meshless local Petrov-Galerkin method. Alibeigloo [9] employed the differential quadrature method (DQM) to derive thermoelasticity solutions for functionally graded solid circular and annular plates integrated with piezoelectric layers. Maslak et al. [10] used the semi-analytical DQM for the 3D thermo-viscoelastic analysis of functionally graded viscoelastic cylindrical panels with embedded piezoelectric layers under asymmetric thermal loading and complex boundary conditions. Yan et al. [11] performed 3D static analysis of FGPMs using the partial differential matrix of the finite block method with Lagrange polynomial interpolation. Van et al. [12] proposed a modified nonlocal strain gradient theory combined with Navier-type semi-analytical solution for the static bending analysis of FGPM nanoplates under electro-mechanical multi-field coupling.

In the theoretical analysis of plate structures, analytical approaches are of great significance and can be broadly divided into simplified plate theories and 3D elasticity theory. Simplified plate theories usually introduce kinematic assumptions. For instance, Nourmohammadi et al. [13] investigated the static response of sandwich FGPM plates based on first-order shear deformation plate theory, in which a normal line remains straight after deformation but is no longer perpendicular to the middle plane. Aniket and Chanda [14] developed an inverse hyperbolic shear deformation theory with five independent variables that inherently satisfies the traction-free boundary conditions on the top and bottom surfaces. They obtained a quasi-exact solution for the bending response, including deflection and stress, of FGPM plates layered with piezoelectric fiber-reinforced composite (PFRC) under electromechanical loads. In addition, He et al. [15] applied a multi-parameter perturbation method based on thin-plate assumptions to investigate FGPM circular plates.

In contrast, analytical solutions derived from 3D elasticity theory, which avoid ad hoc kinematic assumptions, are often used as benchmark solutions for validating two-dimensional (2D) simplified plate theories or 3D approximate numerical or semi-analytical analysis [16]. Zhong and Shang [17] used a state-space approach with the transfer matrix method to obtain exact 3D solutions for simply supported FGPM rectangular plates. Pan and Han [18] derived an exact solution for a three-layered FGPM rectangular plate with simply supported boundary conditions based on the Pseudo-Stroh formalism and the propagator matrix method. Lu et al. [19] presented an alternative Stroh-like derivation for exact solutions of the simply supported FGPM rectangular plate. Zenkour and Hafed [20] analyzed the bending of simply supported FGPM plates using a quasi-3D sinusoidal shear and normal deformation theory with six unknowns. Although these analytical studies mainly focus on rectangular geometries, in FGPM circular plates are also widely used in smart engineering components and pose distinct mathematical challenges. Based on the 3D theory of piezoelectricity, Wang et al. [21] investigated the bending response of FGPM circular plates with thickness-dependent material properties and obtained exact solutions under simply supported and clamped boundary conditions. Wang et al. [22] further derived analytical solutions for multilayered magneto-electro-elastic circular plates using state-space vectors, finite Hankel transforms, and propagating matrix methods, although their approach was limited to simply supported boundary conditions. Yang et al. [23] derived an electroelastic solution for FGPM circular plates subjected to combined mechanical loads, while Li and Gao [24] investigated the axisymmetric free vibration characteristics of such structures. To address more general grading profiles, Li et al. [25,26,27] developed a direct displacement method, derived the explicit displacement function, and provided 3D analytical solutions for transversely isotropic FGPM circular plates under pure bending, uniformly loaded, and uniform electric potential difference. These solutions accommodate arbitrary material variation through the plate thickness.

Because the thickness-dependent material properties of the FGPM plates make the coupled electro-mechanical analyses mathematically complicated, exact analytical solutions are more difficult to obtain than numerical or semi-analytical results. Consequently, research on the problem based on 3D elasticity theory remains quite limited. Although the seminal works [21,22,25,26,27] are based on 3D elasticity, their methodologies still rely on specific expansion assumptions for the displacement and electric fields. The present study therefore proposes a different solution system for the 3D circular plate problem, providing benchmark results for evaluating simplified plate theories and numerical methods.

Mian and Spencer [28] constructed an exact 3D solution for isotropic FGM plates with traction-free surfaces based on 3D elasticity theory, starting from the 2D solution of classical thin plate theory and allowing material parameters to vary arbitrarily and continuously through the thickness. Although the 3D circular plate problem has been investigated in the literature, most existing analytical methods rely on special expansion assumptions for the displacement and electric fields. Our primary innovation lies in methodological improvement, namely, extending the Mian and Spencer solution theory to derive exact analytical solutions for FGPM circular plates. In contrast to these frameworks, the present study constructs an independent electric potential function and successfully extends the Mian and Spencer framework [28] from purely elastic materials to analyze the bending of transversely isotropic FGPM circular plates subjected to a uniform mechanical load. This work represents the first successful extension of the Mian and Spencer plate theory to the 3D FGPM circular plate problem and provides benchmark solutions for the development of simplified plate theory models.

## 2. Basic Equations

**Figure 1:**
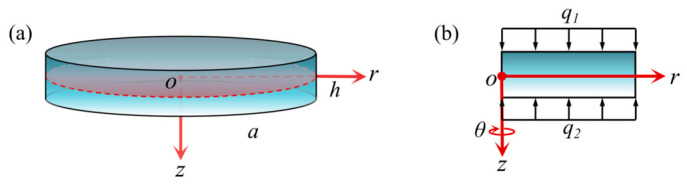
Geometry and the coordinate system of (**a**) an FGPM circular plate and (**b**) the corresponding cross-section.

As illustrated in Figure 1, a transversely isotropic FGPM circular plate with radius a and thickness h is analyzed using a cylindrical coordinate system $\left(r,\theta ,z\right)$, where the origin o is located at the center of the mid-plane, and the *z*-axis is perpendicular to the mid-plane. Uniformly distributed loads $q_{1}$ and $q_{2}$ are applied to its top and bottom surfaces, respectively. Owing to the geometric and loading symmetric, the 3D analysis of the elastic and electric fields can be simplified to an axisymmetric problem. Therefore, the circumferential displacement component vanishes, and all physical fields and material coefficients are independent of the coordinate θ.

Neglecting body forces and electric charge density, the elastic equilibrium equations and Gauss equation of electrostatics are given by [21]:

(1) $$\frac{\partial \sigma_{r}}{\partial r}+\frac{\partial \tau_{rz}}{\partial z}+\frac{\sigma_{r}-\sigma_{\theta }}{r}=0,\;\frac{\partial \sigma_{z}}{\partial z}+\frac{\partial \tau_{rz}}{\partial r}+\frac{\tau_{rz}}{r}=0,\;\frac{\partial D_{r}}{\partial r}+\frac{\partial D_{z}}{\partial z}+\frac{D_{r}}{r}=0,$$

where the stress components are denoted by $\sigma_{r}$, $\sigma_{\theta }$, $\sigma_{z}$ and $\tau_{rz}$, and the electric displacements components are denoted by $D_{z}$, $D_{r}$.

Defining the *z*-axis as both the direction of polarization and the axis of transverse isotropy, the constitutive equations are given by [29]:

(2) $$\sigma_{r}=c_{11}\frac{\partial u_{r}}{\partial r}+c_{12}\frac{u_{r}}{r}+c_{13}\frac{\partial w}{\partial z}+e_{31}\frac{\partial \phi }{\partial z},\;\sigma_{\theta }=c_{12}\frac{\partial u_{r}}{\partial r}+c_{11}\frac{u_{r}}{r}+c_{13}\frac{\partial w}{\partial z}+e_{31}\frac{\partial \phi }{\partial z},\;\sigma_{z}=c_{13}\frac{\partial u_{r}}{\partial r}+c_{13}\frac{u_{r}}{r}+c_{33}\frac{\partial w}{\partial z}+e_{33}\frac{\partial \phi }{\partial z},\;\tau_{rz}=c_{44}\left(\frac{\partial w}{\partial r}+\frac{\partial u_{r}}{\partial z}\right)+e_{15}\frac{\partial \phi }{\partial r},$$

(3) $$D_{r}=e_{15}\left(\frac{\partial w}{\partial r}+\frac{\partial u_{r}}{\partial z}\right)-\varepsilon_{11}\frac{\partial \phi }{\partial r},D_{z}=e_{31}\left(\frac{\partial u_{r}}{\partial r}+\frac{u_{r}}{r}\right)+e_{33}\frac{\partial w}{\partial z}-\varepsilon_{33}\frac{\partial \phi }{\partial z}$$

where $u_{r}$ and w are the displacement components; ϕ is the electric potential; $c_{ij}$, $e_{ij}$ and $\varepsilon_{ij}$ represent the elastic, piezoelectric, and dielectric coefficients, respectively. For the FGPM, these material properties are assumed to vary continuously along the thickness direction and are therefore functions only of the coordinate *z*.

Based on the Mian and Spencer plate theory [28], we adopt the following displacement fields:

(4) $$u_{r}(r,z)={\bar{u}}_{r}(r)+F\left(z\right)\frac{d\Delta }{dr}+A\left(z\right)\frac{d\bar{w}}{dr}+B\left(z\right)\frac{d}{dr}\nabla^{2}\bar{w},\;w(r,z)=\bar{w}\left(r\right)+G\left(z\right)\Delta +C\left(z\right)\nabla^{2}\bar{w}+D\left(z\right),$$

where ${\bar{u}}_{r}\left(r\right)$, $\bar{w}\left(r\right)$ denote the displacement components at the mid-plane, and the remaining terms $A\left(z\right)$, $B\left(z\right)$, $C\left(z\right)$, $D\left(z\right)$,$F\left(z\right)$, $G\left(z\right)$ are unknown thickness-dependent functions to be determined, and

(5) $$\Delta =\frac{d{\bar{u}}_{r}}{dr}+\frac{{\bar{u}}_{r}}{r}=\frac{1}{r}\frac{d}{dr}(r{\bar{u}}_{r}),\;\nabla^{2}=\frac{d^{2}}{dr^{2}}+\frac{1}{r}\frac{d}{dr}=\frac{1}{r}\frac{d}{dr}(r\frac{d}{dr}).$$

Analogous to the displacement expressions defined in Equation (4), the electric potential function is formulated as follows:

(6) $$\phi (r,z)=\phi_{1}\Delta +\phi_{2}\nabla^{2}\bar{w}+\phi_{0},$$

where the corresponding functions are unknown $\phi_{0}\left(z\right)$, $\phi_{1}\left(z\right)$, $\phi_{2}\left(z\right)$ and must be determined from the governing equations and boundary conditions.

Substituting Equations (4)–(6) into the constitutive relations in Equations (2) and (3), we obtain:

(7) $$\begin{array}{cc} \sigma_{r} & =\Delta (c_{11}+c_{13}G^{\prime }+e_{31}{\phi^{\prime }}_{1})+\nabla^{2}\Delta c_{11}F+\nabla^{2}\bar{w}(c_{11}A+c_{13}C^{\prime }+e_{31}{\phi^{\prime }}_{2})+\nabla^{4}\bar{w}c_{11}B\\ & +c_{13}D^{\prime }+e_{31}{\phi^{\prime }}_{0}-2c_{66}\frac{1}{r}({\bar{u}}_{r}+F\Delta_{,r}+A{\bar{w}}_{,r}+B\nabla^{2}{\bar{w}}_{,r}),\\ \sigma_{\theta } & =\Delta (c_{\;12}+c_{13}G^{\prime }+e_{31}{\phi^{\prime }}_{1})+c_{12}F\nabla^{2}\Delta +\nabla^{2}\bar{w}(c_{12}A+c_{13}C^{\prime }+e_{31}{\phi^{\prime }}_{2})+\nabla^{4}\bar{w}c_{12}B\;\\ & +c_{13}D^{\prime }+e_{31}{\phi^{\prime }}_{0}+2c_{66}\frac{1}{r}({\bar{u}}_{r}+F\Delta_{,r}+A{\bar{w}}_{,r}+B\nabla^{2}{\bar{w}}_{,r}),\\ \sigma_{z} & =\Delta (c_{\;13}+c_{33}G^{\prime }+e_{33}{\phi^{\prime }}_{1})+\nabla^{2}\bar{w}(c_{13}A+c_{33}C^{\prime }+e_{33}{\phi^{\prime }}_{2})+\nabla^{2}\Delta c_{13}F+\nabla^{4}\bar{w}c_{13}B\\ & +c_{33}D^{\prime }+e_{33}{\phi^{\prime }}_{0}\;, \end{array}\;\tau_{\mathrm{rz}}=\Delta_{,r}[c_{44}(G+F^{\prime })+e_{15}\phi_{1}]+\nabla^{2}{\bar{w}}_{,r}[c_{44}(C+B^{\prime })+e_{15}\phi_{2}]\;+{\bar{w}}_{,r}c_{44}(A^{\prime }+1)$$

(8) $$\begin{array}{cc} D_{r} & =\Delta_{,r}[e_{15}(G+F^{\prime })-\varepsilon_{11}\phi_{1}]+\nabla^{2}{\bar{w}}_{,r}[e_{15}(C+B^{\prime })-\varepsilon_{11}\phi_{2}]+{\bar{w}}_{,r}e_{15}(A^{\prime }+1),\\ D_{z} & =\Delta (e_{31}+e_{33}G^{\prime }-\varepsilon_{33}{\phi^{\prime }}_{1})+\nabla^{2}\Delta e_{31}F+\nabla^{2}\bar{w}(e_{31}A+e_{33}C^{\prime }-\varepsilon_{33}{\phi^{\prime }}_{2})\\ & \;+\nabla^{4}\bar{w}e_{31}B+e_{33}D^{\prime }-\varepsilon_{33}{\phi^{\prime }}_{0}. \end{array}$$

in which the prime symbol “′” denotes differentiation with respect to *z*, and “,” denotes the partial derivative with respect to the radial coordinate r.

By substituting Equations (7) and (8) into Equation (1), the 3D equilibrium equations are transformed into a set of ordinary differential equations with respect to the thickness coordinate *z*. Equation (1) can be satisfied merely by solving the following equations for the unknown *z*-dependent functions:

(9) $${\left[c_{44}(A^{\prime }+1)\right]}^{\prime }=0,$$

(10) $$(c_{13}+c_{33}G^{\prime }+e_{33}{\phi^{\prime }}_{1}{)}^{\prime }=0,$$

(11) $$(e_{31}+e_{33}G^{\prime }-\varepsilon_{33}{\phi^{\prime }}_{1}{)}^{\prime }=0,$$

(12) $$c_{44}(A^{\prime }+1)+(c_{13}A+c_{33}C^{\prime }+e_{33}{\phi^{\prime }}_{2}{)}^{\prime }=0,$$

(13) $$e_{15}(A^{\prime }+1)+(e_{31}A+e_{33}C^{\prime }-\varepsilon_{33}{\phi^{\prime }}_{2}{)}^{\prime }=\nabla^{2}\bar{w},$$

(14) $$c_{11}+c_{13}G^{\prime }+e_{31}{\phi^{\prime }}_{1}+[c_{44}(G+F^{\prime })+e_{15}\phi_{1}{]}^{\prime }=c_{44}\kappa_{1},$$

(15) $$c_{11}A+c_{13}C^{\prime }+e_{31}{\phi^{\prime }}_{2}+[c_{44}(C+B^{\prime })+e_{15}\phi_{2}{]}^{\prime }=c_{44}\kappa_{2},$$

(16) $$\nabla^{2}\Delta =\kappa_{3},$$

(17) $$\nabla^{4}\bar{w}=\kappa_{4},$$

where the constants appearing in these equations are arbitrary integration constants.

Substituting Equation (5) into Equations (16) and (17) gives

(18) $$\frac{1}{r}\frac{\partial }{\partial r}\left(r\frac{\partial \Delta }{\partial r}\right)=\kappa_{3},\;\nabla^{2}\left(\nabla^{2}\bar{w}\right)=\kappa_{4}.$$

Integrating the above two equations with respect to the radial coordinate r yields:

(19) $$\Delta_{r}=\frac{\kappa_{3}}{2}r+\frac{P_{1}}{r},{\left(\nabla^{2}\bar{w}\right)}_{,r}=\frac{\kappa_{4}}{2}r+\frac{P_{2}}{r}.$$

where $P_{1}$ and $P_{2}$ are integration constants.

Accordingly, Equation (1) can be rewritten as the following set of equations:

(20) $$\kappa_{1}\Delta_{,r}+\kappa_{2}\nabla^{2}{\bar{w}}_{,r}=0,$$

(21) $$\kappa_{3}[c_{44}(G+F^{\prime })+e_{15}\phi_{1}+(c_{13}F{)}^{\prime }]+\kappa_{4}[c_{44}(C+B^{\prime })+e_{15}\phi_{2}+(c_{13}B{)}^{\prime }]+(c_{33}D^{\prime }+e_{33}{\phi^{\prime }}_{0}{)}^{\prime }=0,$$

(22) $$\kappa_{3}[e_{15}(G+F^{\prime })-\varepsilon_{11}\phi_{1}+(e_{31}F{)}^{\prime }]+\kappa_{4}[e_{15}(C+B^{\prime })-\varepsilon_{11}\phi_{2}+(e_{31}B{)}^{\prime }]+(e_{33}D^{\prime }-\varepsilon_{33}{\phi^{\prime }}_{0}{)}^{\prime }=0.$$

Substituting Equation (19) into Equation (20) leads to:

(23) $$\frac{1}{2}\left(\kappa_{1}\kappa_{3}+\kappa_{2}\kappa_{4}\right)+r^{-1}\left(\kappa_{1}P_{1}+\kappa_{2}P_{2}\right)=0.$$

For Equation (23) to hold for any arbitrary radial coordinate *r*, the coefficients of the independent radial terms must vanish separately. Therefore, the following condition is obtained:

(24) $$\kappa_{1}\kappa_{3}+\kappa_{2}\kappa_{4}=0.$$

By solving the differential equations presented in Equations (17) and (20), the explicit expressions for the mid-plane displacement components are derives as follows:

(25) $$\bar{w}(r)=\frac{\kappa_{4}}{64}r^{4}+C_{1}r^{2}+C_{2},\;\bar{u}(r)=\frac{\kappa_{3}}{16}r^{3}-\frac{2\kappa_{2}}{\kappa_{1}}C_{1}r+\frac{1}{2\kappa_{1}}C_{3}r.$$

when z=0, it follows from Equations (4) and (6) that $A\left(0\right)$ = $B\left(0\right)$ = $C\left(0\right)$ = $D\left(0\right)$ = $F\left(0\right)$ = $G\left(0\right)$ = 0.

## 3. FGPM Circular Plates Subject to a Uniform Load

### 3.1. Determination of Displacement and Potential Functions

For an FGPM circular plate subjected to uniform transverse loads, the mechanical and electrical boundary conditions on the top $\left(z=h/2\right)$ and bottom $\left(z=-h/2\right)$ surfaces are prescribed as follows:

(26) $$z=-\frac{h}{2}:\;\;\;\;\;\sigma_{z}=-q_{1},\;\;\;\;\tau_{rz}=0,\;\;\;\;D_{z}=0.$$

(27) $$z=\frac{h}{2}:\;\;\;\;\;\sigma_{z}=-q_{2},\;\;\;\;\tau_{rz}=0,\;\;\;\;D_{z}=0.$$

Substituting the relevant stress and electric displacement components ($\sigma_{z}$, $\tau_{rz}$ and $D_{z}$) from Equations (7) and (8) into the boundary conditions in Equations (26) and (27) leads to

(28) $$A^{\prime }(\pm h/2)+1=0,$$

(29) $$c_{13}(\pm h/2)+c_{33}(\pm h/2)G^{\prime }(\pm h/2)+e_{33}(\pm h/2){\phi^{\prime }}_{1}(\pm h/2)=0,$$

(30) $$c_{13}(\pm h/2)A(\pm h/2)+c_{33}(\pm h/2)C^{\prime }(\pm h/2)+e_{33}(\pm h/2){\phi^{\prime }}_{2}(\pm h/2)=0,$$

(31) $${\left\{c_{13}(z)[\kappa_{3}F(z)+\kappa_{4}B(z)]+c_{33}(z)D^{\prime }(z)+e_{33}(z){\phi^{\prime }}_{0}(z)\right\}}_{z=-h/2}=-q_{1},$$

(32) $${\left\{c_{13}(z)[\kappa_{3}F(z)+\kappa_{4}B(z)]+c_{33}(z)D^{\prime }(z)+e_{33}(z){\phi^{\prime }}_{0}(z)\right\}}_{z=h/2}=-q_{2},$$

(33) $$c_{44}(\pm h/2)[G(\pm h/2)+F^{\prime }(\pm h/2)]+e_{15}(\pm h/2)\phi_{1}(\pm h/2)=0,$$

(34) $$c_{44}(\pm h/2)[C(\pm h/2)+B^{\prime }(\pm h/2)]+e_{15}(\pm h/2)\phi_{2}(\pm h/2)=0,$$

(35) $$e_{31}(\pm h/2)+e_{33}(\pm h/2)G^{\prime }(\pm h/2)-\varepsilon_{33}(\pm h/2){\phi^{\prime }}_{1}(\pm h/2)=0,$$

(36) $$e_{31}(\pm h/2)A(\pm h/2)+e_{33}(\pm h/2)C^{\prime }(\pm h/2)-\varepsilon_{33}(\pm h/2){\phi^{\prime }}_{2}(\pm h/2)=0,$$

(37) $${\left\{e_{31}(z)[\kappa_{3}F(z)+\kappa_{4}B(z)]+e_{33}(z)D^{\prime }(z)-\varepsilon_{33}(z){\phi^{\prime }}_{0}(z)\right\}}_{z=\pm h/2}=0.$$

Integrating Equations (9)–(15), (21) and (22) and using Equations (28)–(31) and (33)–(35), yield the displacement and potential functions in Equations (4) and (6):

(38) $$A\left(z\right)=-z+A_{0},\;\;C(z)=C_{1}(z)+C_{0},\;\;G(z)=G_{1}(z)+G_{0},$$

(39) $$F(z)=\int_{-h/2}^{z}\frac{F_{11}\left(z\right)-e_{15}\phi_{1}\left(z\right)}{c_{44}}-Gdz+F_{0},\;B(z)=\int_{-h/2}^{z}\frac{B_{11}\left(z\right)-e_{15}\phi_{2}\left(z\right)}{c_{44}}-Cdz+B_{0},$$

(40) $$D(z)=\int_{-h/2}^{z}\frac{-\varepsilon_{33}\left[D_{01}\left(z\right)+D_{11}\right]-e_{33}\left[D_{02}\left(z\right)+D_{22}\right]}{e_{33}^{2}+c_{33}\varepsilon_{33}}dz+D_{0},$$

(41) $$\phi_{0}(z)=\int_{-h/2}^{z}\frac{-e_{33}\left[D_{01}\left(z\right)+D_{11}\right]+c_{33}\left[D_{02}\left(z\right)+D_{22}\right]}{e_{33}^{2}+c_{33}\varepsilon_{33}}dz+\Phi_{0},$$

(42) $$\phi_{1}(z)=\phi_{10}\left(z\right)+\Phi_{1},\;\;\phi_{2}(z)=\phi_{20}\left(z\right)+\Phi_{2}.$$

where the integration constants $A_{0}$, $B_{0}$, $C_{0}$, $D_{0}$, $F_{0}$ and $G_{0}$ are provided in Appendix A.

### 3.2. Determination of Remaining Integral Constants

Integrating Equations (14) and (15) over the plate thickness and invoking the boundary conditions in Equations (33) and (31) yields:

(43) $$\kappa_{1}=\int_{-h/2}^{h/2}\left(c_{11}+c_{13}G^{\prime }+e_{31}{\phi^{\prime }}_{1}\right)dz/\int_{-h/2}^{h/2}c_{44}dz,\;\kappa_{2}=-\int_{-h/2}^{h/2}\left(c_{11}A+c_{13}C^{\prime }+e_{31}{\phi^{\prime }}_{2}\right)dz/\int_{-h/2}^{h/2}c_{44}dz,$$

Integrating Equation (21) and using Equations (31) and (32), we get

(44) $$\kappa_{3}\kappa_{31}+\kappa_{4}\kappa_{41}=q_{2}-q_{1},$$

where

(45) $$\kappa_{31}=\int_{-h/2}^{h/2}\left\{c_{44}\left[G\left(z\right)+F^{\prime }\left(z\right)\right]+e_{15}\phi_{1}\right\}dz,\;\kappa_{41}=\int_{-h/2}^{h/2}\left\{c_{44}\left[C\left(z\right)+B^{\prime }\left(z\right)\right]+e_{15}\phi_{2}\right\}dz.$$

Substituting Equation (42) into Equation (45) gives the following updated expression:

(46) $$\kappa_{31}=\int_{-h/2}^{h/2}\kappa_{31}^{*}\left(z\right)dz+\Phi_{1}\int_{-h/2}^{h/2}e_{15}dz,\;\;\kappa_{41}=\int_{-h/2}^{h/2}\kappa_{41}^{*}\left(z\right)dz+\Phi_{2}\int_{-h/2}^{h/2}e_{15}dz,$$

where

(47) $$\kappa_{31}^{*}\left(z\right)=c_{44}\left[G\left(z\right)+F^{\prime }\left(z\right)\right]+e_{15}\phi_{10}\left(z\right),\;\kappa_{41}^{*}\left(z\right)=c_{44}\left[C\left(z\right)+B^{\prime }\left(z\right)\right]+e_{15}\phi_{20}\left(z\right).$$

Integrating Equation (22) and using Equation (37), we obtain:

(48) $$\kappa_{3}\kappa_{32}+\kappa_{4}\kappa_{42}=0,$$

where

(49) $$\kappa_{32}=\int_{-h/2}^{h/2}\left\{c_{44}\left[G\left(z\right)+F^{\prime }\left(z\right)\right]-\varepsilon_{11}\phi_{1}\left(z\right)\right\}dz,\;\kappa_{42}=\int_{-h/2}^{h/2}\left\{c_{44}\left[C\left(z\right)+B^{\prime }\left(z\right)\right]-\varepsilon_{11}\phi_{2}\left(z\right)\right\}dz.$$

Consequently, the constants $\kappa_{3}$ and $\kappa_{4}$ can be determined by solving Equations (44) and (48) simultaneously:

(50) $$\kappa_{3}=\frac{\kappa_{42}\left(q_{2}-q_{1}\right)}{\kappa_{31}\kappa_{42}-\kappa_{41}\kappa_{32}},\;\kappa_{4}=\frac{\kappa_{32}\left(q_{1}-q_{2}\right)}{\kappa_{31}\kappa_{42}-\kappa_{41}\kappa_{32}}.$$

Assuming the displacement at z=0 corresponds to the mid-plane displacement, Equation (4) dictates that functions $A\left(z\right)∼G\left(z\right)$ vanish at z=0. For physical consistency, adopting z=0 as the reference electric potential naturally yields $\phi_{1}\left(0\right)=0$ and $\phi_{0}\left(0\right)=0$. Consequently, substituting Equations (41) and (42) into Equation (6) determines $\Phi_{1}$ and $\Phi_{2}$. As an arbitrary reference potential, $\Phi_{0}$ does not affect the electro-elastic field. The complete expressions of these constants are provided in Appendix A.

### 3.3. Radial Resultant Force and Bending Moment

By utilizing the radial stress ${\bar{\sigma }}_{r}$ provided in Equation (7), the expressions of the radial resultant force $N\left(r\right)$ and bending moment $M\left(r\right)$ can be obtained:

(51) $$\begin{array}{c} N(r)\equiv \int_{-h/2}^{h/2}\sigma_{r}dz=\Delta N_{1}+\nabla^{2}\bar{w}N_{3}+\nabla^{2}\Delta N_{5}+\nabla^{4}\bar{w}N_{7}+N_{0}\\ -\frac{2}{r}\left({\bar{u}}_{r}N_{9}+\Delta_{,r}N_{11}+{\bar{w}}_{,r}N_{13}+\nabla^{2}{\bar{w}}_{,r}N_{15}\right), \end{array}$$

(52) $$\begin{array}{c} M(r)\equiv \int_{-h/2}^{h/2}\sigma_{r}zdz=\Delta M_{1}+\nabla^{2}\bar{w}M_{3}+\nabla^{2}\Delta M_{5}+\nabla^{4}\bar{w}M_{7}+M_{0}\\ -\frac{2}{r}\left({\bar{u}}_{r}M_{9}+\Delta_{,r}M_{11}+{\bar{w}}_{,r}M_{13}+\nabla^{2}{\bar{w}}_{,r}M_{15}\right), \end{array}$$

where

(53) $$\begin{array}{l} N_{0}=\int_{-h/2}^{h/2}(c_{13}D^{\prime }+e_{31}{\phi^{\prime }}_{1})dz,\;\;N_{1}=\int_{-h/2}^{h/2}(c_{11}+c_{13}G^{\prime }+e_{31}{\phi^{\prime }}_{1})dz,\\ N_{3}=\int_{-h/2}^{h/2}(c_{11}A+c_{13}C^{\prime }+e_{31}{\phi^{\prime }}_{2})dz,\;\;N_{5}=\int_{-h/2}^{h/2}c_{11}Fdz,\;\;N_{7}=\int_{-h/2}^{h/2}c_{11}Bdz,\\ N_{9}=\int_{-h/2}^{h/2}c_{66}dz,\;\;N_{11}=\int_{-h/2}^{h/2}c_{66}Fdz,\;\;N_{13}=\int_{-h/2}^{h/2}c_{66}Adz,\;\;N_{15}=\int_{-h/2}^{h/2}c_{66}Bdz,\\ M_{0}=\int_{-h/2}^{h/2}z(c_{13}D^{\prime }+e_{31}{\phi^{\prime }}_{0})dz,\;M_{1}=\int_{-h/2}^{h/2}z(c_{11}+c_{13}G^{\prime }+e_{31}{\phi^{\prime }}_{1})dz,\\ M_{3}=\int_{-h/2}^{h/2}z(c_{11}A+c_{13}C^{\prime }+e_{31}{\phi^{\prime }}_{2})dz,\;M_{5}=\int_{-h/2}^{h/2}z(c_{11}F)dz,\;M_{7}=\int_{-h/2}^{h/2}z(c_{11}B)dz,\\ M_{9}=\int_{-h/2}^{h/2}zc_{66}dz,\;M_{11}=\int_{-h/2}^{h/2}zc_{66}Fdz,\;M_{13}=\int_{-h/2}^{h/2}zc_{66}Adz,\;M_{15}=\int_{-h/2}^{h/2}zc_{66}Bdz. \end{array}$$

In this study, three integral constants $C_{i}(i=1,2,3)$ can be determined by applying the boundary conditions at the edge of the FGPM circular plate (r=a). Specifically, we investigate one simply supported (SS) and two types of clamped (C1 and C2) boundary conditions, which are mathematically defined as follows:

(54) $$\mathrm{SS}:\;\;\;\;\;N_{r}(a)=0,\;\;\;\;\bar{w}(a,0)=0,\;\;\;\;M_{r}(a)=0.$$

(55) $$C1:\;\;\;\;\;\bar{u}(a,0)=0,\;\;\;\;\bar{w}(a,0)=0,\;\;\;\;{\bar{w}}_{,r}(a,0)=0.$$

(56) $$C2:\;\;\;\;\;\bar{u}(a,0)=0,\;\;\;\;\bar{w}(a,0)=0,\;\;\;\;u_{,z}(a,0)=0.$$

It should be noted that, in the present analytical framework, the pointwise boundary conditions on the cylindrical edge are replaced by statically equivalent resultant forces. This treatment follows Saint-Venant’s principle and facilitates the derivation of exact 3D elasticity solutions. Consequently, the proposed solution is particularly suitable for moderately thick circular plates (β≈0.15), for which local edge effects decay rapidly away from the boundary.

Once these integration constants are determined, the complete exact 3D elasticity solutions for the electro-elastic fields of uniformly loaded FGPM circular plates can be obtained from Equations (4)–(8).

## 4. Numerical Results and Discussions

Unless otherwise specified, the numerical examples presented herein consider an FGPM circular plate with a radius a=0.1 m and a thickness-to-radius ratio β=0.1. The plate is subjected to uniformly distributed mechanical loads on the top and bottom surfaces, defined as $q_{1}=1\times 10^{6}$ N/m^2^ and $q_{2}=0$ N/m^2^, respectively. To facilitate the analysis, the following dimensionless parameters are introduced:

(57) $${\bar{\sigma }}_{r}=\frac{\sigma_{r}}{q_{1}},\;\;\;\;{\bar{\sigma }}_{z}=\frac{\sigma_{z}}{q_{1}},\;\;\;\;{\bar{\tau }}_{rz}=\frac{\tau_{rz}}{q_{1}},\;\;\;\;\bar{W}=\frac{w}{h},\;\;\;\;W=\frac{\beta^{3}wc_{11}^{PZT}}{q_{1}h},\;\bar{U}=\frac{u_{r}}{a},\;\;\;\;{\bar{D}}_{r}=\frac{D_{r}}{\sqrt{c_{33}\varepsilon_{33}}},\;\;\;\;D_{z}=\frac{D_{z}}{\sqrt{c_{33}\varepsilon_{33}}},\;\;\;\;\bar{\Phi }=\frac{\phi }{h}\sqrt{\frac{\varepsilon_{33}}{c_{33}}},\;\;\;\;\beta =\frac{h}{a}.$$

### 4.1. Verification and Comparison

To verify the solutions obtained by the present method, an FGPM circular plate subjected to a uniformly distributed load is considered, with its material constants given by the following expressions:

(58) $$c_{ij}\left(z\right)=\left(1-0.2\left(\frac{z}{h}+0.5\right)\gamma_{ij}^{c}\right)c_{ij}^{PZT}+0.2\frac{z}{h}c_{ij}^{Pt},\;e_{ij}\left(z\right)=\left(1-0.2\left(\frac{z}{h}+0.5\right)\gamma_{ij}^{e}\right)e_{ij}^{PZT},\;\;\varepsilon_{ij}\left(z\right)=\left[1+0.9{\left(\frac{z}{h}+0.5\right)}^{2}\gamma_{ij}^{\varepsilon }\right]\varepsilon_{ij}^{PZT},$$

where the superscripts PZT and Pt denote the piezoelectric ceramic and the metallic platinum materials, respectively. Their material parameters can be found in Table 1 [27], while $\gamma_{ij}^{c}$, $\gamma_{ij}^{e}$ and $\gamma_{ij}^{\varepsilon }$ serve as dimensionless material parameters.

When $\gamma_{ij}^{c}$, $\gamma_{ij}^{e}$ and $\gamma_{ij}^{\varepsilon }$ are taken as 1, Table 2 presents the dimensionless central deflection $\bar{W}$ of the simply supported FGPM circular plate. The comparative results demonstrate that the present exact 3D analytical solutions are in good agreement with those reported by Wang et al. [21] and Li et al. [27], both of which are based on elasticity theory.

Furthermore, by adopting the specific parameter set $\gamma_{11}^{c}=0$, $\gamma_{12}^{c}=0.25$, $\gamma_{13}^{c}=\gamma_{15}^{e}=\gamma_{11}^{\varepsilon }=0.5$ and $\gamma_{33}^{c}=\gamma_{31}^{e}=0.75$, Table 3 depicts a comparison between the present results and those obtained by Li et al. [27] with β=0.3 and r=0. As shown in the table, the maximum relative errors for ${\bar{\sigma }}_{z}$ and ${\bar{\sigma }}_{r}$ are merely 0.78% and 0.83%, respectively. The solutions from the two methods show excellent agreement, thereby verifying the accuracy of the present analytical solution.

### 4.2. Parameter Analysis

To further investigate the electro-elastic responses of FGPMs, we consider a circular FGPM plate with the material coefficients exponentially graded along the thickness direction:

(59) $$M_{ij}(z)=M_{ij}^{0}e^{\lambda \left(\frac{z}{h}+0.5\right)},\;\left(i,j=1,2,3,4,5\right),$$

where $M_{ij}^{0}$ represents material properties of the PZT-4 at z=−h/2 and are given in Table 4. It is obvious that λ=0 corresponds to a homogeneous piezoelectric circular plate. On this basis, the influences of the material gradient index, thickness-to-radius ratio, boundary conditions and piezoelectric effect on the electro-elastic responses of the circular plate are analyzed systematically.

#### 4.2.1. Influence of the Material Gradient Index

**Figure 2:**
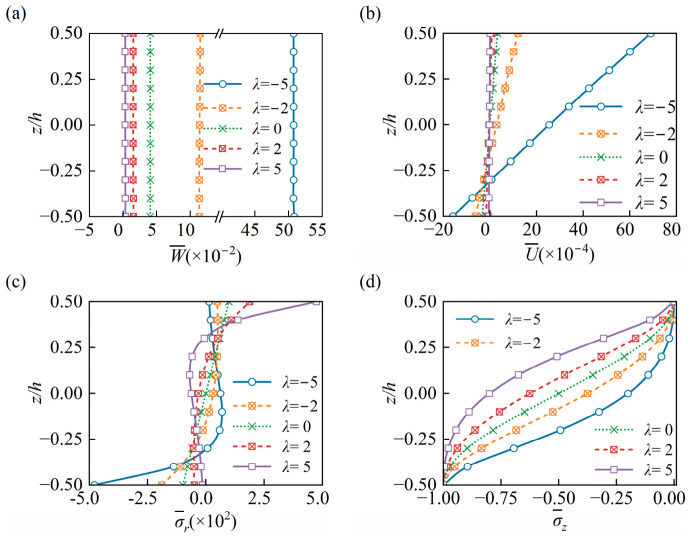

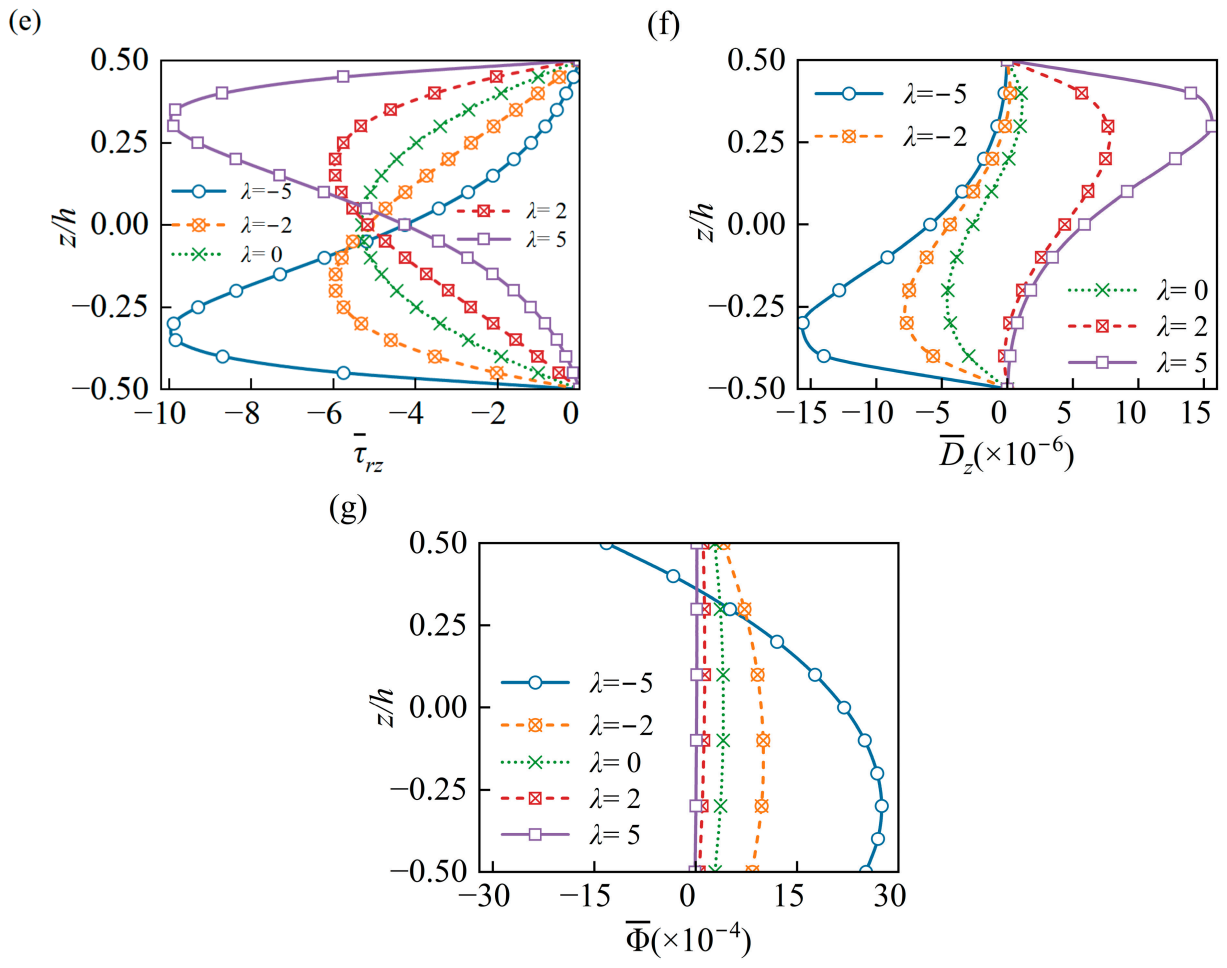
Effects of gradient index on dimensionless physical quantities along the thickness of the plate (r=a/2, β=0.1): (**a**) axial displacement $\bar{W}$; (**b**) radial displacement $\bar{U}$; (**c**) radial stress ${\bar{\sigma }}_{r}$; (**d**) axial stress ${\bar{\sigma }}_{z}$; (**e**) shear stress ${\bar{\tau }}_{rz}$; (**f**) electric displacement component ${\bar{D}}_{z}$; (**g**) electric potential $\bar{\Phi }$.

Figure 2 presents the through-thickness distributions of several dimensionless physical quantities for simply supported FGPM circular plates with different gradient index λ. The distribution of the axial displacement $\bar{W}$ in Figure 2a is almost constant along the thickness direction, which confirms the classical plate theory that the transverse deflection remains nearly unchanged through the thickness. The magnitude of $\bar{W}$ decreases as the gradient index λ increases, because the overall stiffness of the circular plate is enhanced. In Figure 2b, the in-plane displacement $\bar{U}$ varies almost linearly through the thickness and reaches its maximum absolute value on the top surface. As shown in Figure 2c, the in-plane stress component ${\bar{\sigma }}_{r}$ is linear for the homogeneous piezoelectric material (when λ=0) but becomes nonlinear for FGPMs. For positive and negative values of λ, the maximum tensile and compressive stresses occur at the top and bottom surfaces, respectively. The out-of-plane stress components and electric displacement shown in Figure 2d–f also vary nonlinearly through the thickness and strictly satisfy the prescribed mechanical and electrical boundary conditions on the top and bottom surfaces. Finally, Figure 2g shows that the electric potential $\bar{\Phi }$ has an approximately parabolic through-thickness distribution. For homogeneous materials, the electric potential is symmetric about the mid-plane. Gradient variations significantly affect the electric potential distribution in the bottom half of the plate (*z*/*h* = −0.5~0.0), but have a weaker influence in the top half (*z*/*h* = 0.5~0.0).

#### 4.2.2. Influence of the Boundary Condition

**Figure 3:**
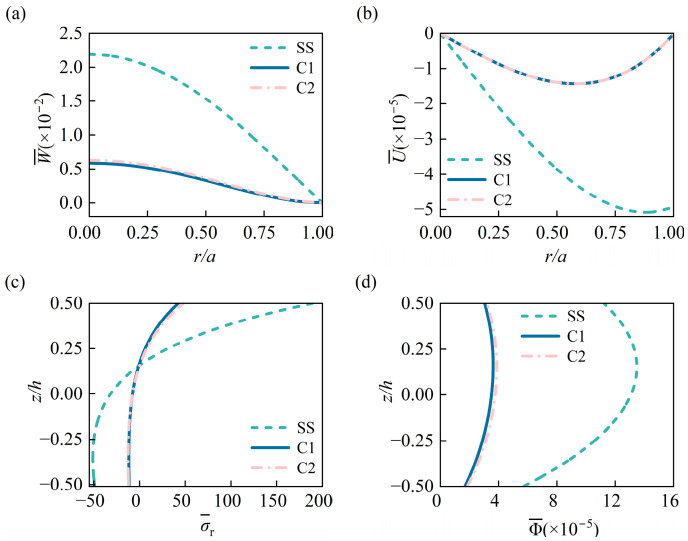
Effects of cylindrical boundary condition on the dimensionless physical quantities along the radial (z/h=0) and thickness (r=a/2) of the plate (λ=2, β=0.1): (**a**) axial displacement $\bar{W}$; (**b**) radial displacement $\bar{U}$ (z=0); (**c**) radial stress ${\bar{\sigma }}_{r}$; (**d**) electric potential $\bar{\Phi }$.

Figure 3 shows the distributions of dimensionless displacement components $\bar{W}$ and $\bar{U}$ along the radial direction at the mid-plane, as well as the dimensionless radial stress ${\bar{\sigma }}_{r}$ and electric potential $\bar{\Phi }$ through the thickness, for three different types of boundary conditions (SS, C1 and C2). It is obvious that there is only a slight difference between the elastic and electric fields for the two types of the clamped boundary conditions (C1 and C2). Furthermore, while the specific type of the boundary conditions (simply supported and clamped) does not alter their distribution patterns of these fields, it significantly influences their magnitudes. As shown in Figure 3a,b, the absolute values of the displacement components $\bar{W}$ and $\bar{U}$ for the same point within the FGPM circular plate under the simply supported boundary condition are significantly higher than those under the clamped boundary conditions. This is because the clamped edges impose stronger kinematic constraints, which enhance the effective flexural rigidity of the plate and consequently suppress the displacement response. Similarly, it can be found in Figure 3c,d that the ${\bar{\sigma }}_{r}$ and $\bar{\Phi }$ curves corresponding to the simply supported boundary condition exhibit more pronounced nonlinear characteristics compared to those under the clamped boundary conditions.

#### 4.2.3. Influence of Thickness-to-Radius Ratio

**Figure 4:**
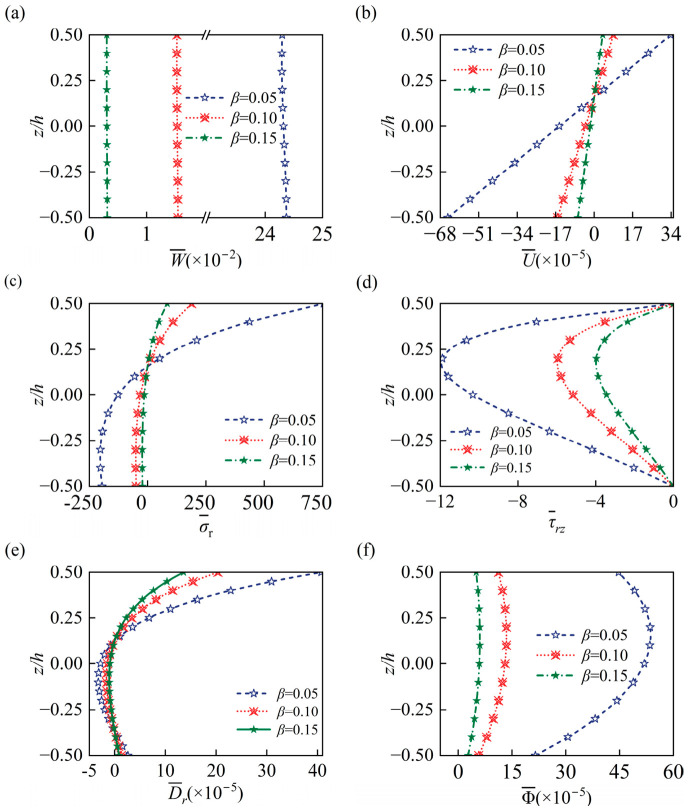
Effects of thickness-to-radius ratio on dimensionless physical quantities along the thickness of the plate (r=a/2, λ=2): (**a**) axial displacement $\bar{W}$; (**b**) radial displacement $\bar{U}$; (**c**) radial stress ${\bar{\sigma }}_{r}$; (**d**) shear stress ${\bar{\tau }}_{rz}$; (**e**) radial electrical displacement ${\bar{D}}_{r}$; (**f**) electric potential $\bar{\Phi }$.

To investigate the effect of the thickness-to-radius ratio on the electro-elastic responses, the thickness of the FGPM circular plate is fixed as *h* = 10 cm, while the thickness-to-radius *β* is taken as 0.05, 0.10 and 0.15 by adjusting the radius a. In accordance with Saint-Venant’s principle and the statically equivalent edge conditions discussed in Section 3.3, the maximum thickness-radius ratio *β* is limited to 0.15 so that the plate remains moderately thick and local edge effects decay rapidly. Figure 4 illustrates the through-thickness distributions of dimensionless physical quantities for simply supported FGPM circular plates with different *β*. Similar to the effect of the boundary conditions, changing this ratio does not alter the general distribution patterns of the field variables. As *β* increases from 0.05 to 0.15, the bending stiffness of the circular plate increases, causing corresponding changes in the magnitude of the physical quantities. Figure 4a,b show that the out-of-plane and in-plane displacement components ($\bar{W}$ and $\bar{U}$) are nearly constant and nearly linear, respectively. The absolute values of both $\bar{W}$ and $\bar{U}$ decrease as *β* increases. In addition, the stress components ${\bar{\sigma }}_{r}$ and ${\bar{\tau }}_{rz}$, electric displacement ${\bar{D}}_{r}$ and electric potential $\bar{\Phi }$ in Figure 4c–f exhibit increasingly pronounced nonlinear variations as *β* increases.

#### 4.2.4. Influence of Piezoelectric Effect

**Figure 5:**
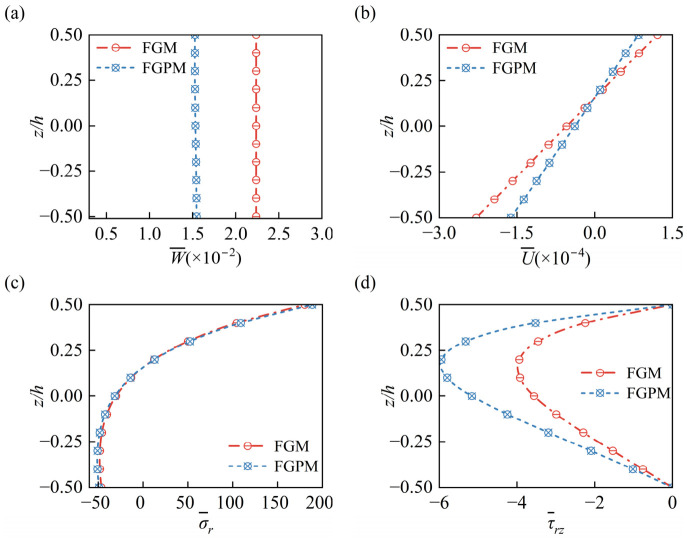

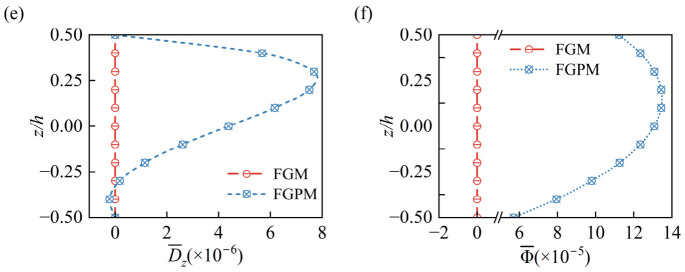
Effects of the piezoelectric effect on dimensionless physical quantities along the thickness of the plate (r=a/2, β=0.1, λ=2): (**a**) axial displacement $\bar{W}$; (**b**) radial displacement; (**c**) radial stress ${\bar{\sigma }}_{r}$; (**d**) shear stress ${\bar{\tau }}_{rz}$; (**e**) axial electrical displacement ${\bar{D}}_{z}$; (**f**) electric potential $\bar{\Phi }$.

Figure 5 illustrates the through-thickness distributions of dimensionless physical quantities for a simply supported FGPM circular plate. To isolate the piezoelectric effect, the results for a purely elastic FGM circular plate, obtained by neglecting the piezoelectric coefficients, are included as a control group. Figure 5a,b,d show that the maximum values of the displacement components ($\bar{W}$ and $\bar{U}$) decrease by approximately 31.92% and 30.77% when piezoelectric effect is consideration, whereas the absolute maximum value of the shear stress ${\bar{\tau }}_{rz}$ increases by 57.15%. As for the radial stress ${\bar{\sigma }}_{r}$ in Figure 5c, the influence of the piezoelectric effect is negligible. Figure 5e,f show that when the piezoelectric effect is included, ${\bar{D}}_{z}$ and $\bar{\Phi }$ exhibit distinctly nonlinear distributions along the thickness direction.

From an engineering application perspective, the substantial increase in shear stress induced by the piezoelectric effect deserves critical attention in structural design. The present results indicate that the peak ${\bar{\tau }}_{rz}$ increases by 57.15% when electro-mechanical coupling is considered, which may increase the risk of interlaminar shear failure, delamination, and premature damage in FGPM circular plates under service loads. Although the radial normal stress is almost unaffected by electro-mechanical coupling, the shear stress is highly sensitive to the piezoelectric effect because of the coupling terms between strain and electric field in the linear piezoelectric constitutive equations. Neglecting the piezoelectric effect may therefore lead to severe underestimation of shear loads and potentially unsafe structural designs for FGPM plates.

## 5. Conclusions

In this paper, the electro-elastic response of an FGPM circular plates subjected to a uniformly distributed load is investigated. The governing equations are derived by extending the Mian and Spencer plate theory, in which an independent electric potential function is constructed by analogy with the mechanical displacement components. As a result, 3D elasticity solutions are obtained for the FGPM circular plate, with the material constants varying arbitrarily along the thickness direction. The present solutions agree well with the available 3D analytical results. The main conclusions are summarized as follows:

1. The material gradient index changes both the through-thickness distribution patterns and magnitudes of the displacement, stress and electric variables. This confirms that material inhomogeneity provides an effective mechanism for tuning the electro-elastic behavior of FGPM circular plates;
2. For the same geometric and loading conditions, the displacement, stress and electric variables of the simply supported FGPM circular plate are generally larger than those of the clamped circular plates;
3. As the thickness-to-radius ratio increases, the stiffness of the FGPM circular plate increases, resulting in lower displacement, stress, and electric-variable magnitudes. Because the pointwise boundary conditions on the cylindrical surface are replaced by statically equivalent resultant forces according to Saint-Venant’s principle, the present theory is most suitable for moderately thick plates (*β* ≈ 0.15).
4. The electro-mechanical coupling intrinsic to FGPMs plays a critical role in structural behavior. Compared with FGM circular plates, considering the piezoelectric effect reduces the maximum transverse deflection by up to 31.92%. However, the absolute maximum shear stress is increased by 57.15%, which may have adverse effects and should therefore be considered carefully in engineering design. These findings demonstrate the importance of electro-elastic coupling in tailoring the responses of FGPM circular plates.

The present 3D elasticity solutions can also serve as benchmark results for evaluating simplified plate theories and numerical methods for the problem studied in this paper.

## Figures and Tables

**Table 1:** Material properties of PZT-4 [27].

Property	PZT-4				
Elastic (GPa)	$c_{11}=120$	$c_{12}=75.2$	$c_{13}=75.1$	$c_{33}=111$	$c_{44}=21.1$
Piezoelectric (C/m^2^)	$e_{15}=12.3$	$e_{31}=-5.35$	$e_{33}=15.8$		
Dielectric (10^−9^ F/m)	$\varepsilon_{11}=8.14$	$\varepsilon_{33}=7.32$			

**Table 2:** Variation in dimensionless deflection $W=\beta^{3}wc_{11}^{PZT}/q_{1}h$ at plate center.

*β*	Wang et al. [21]	Li et al. [27]	Present
0.01	81.949	81.945	81.927
0.05	16.430	16.428	16.425
0.10	8.277	8.276	8.232
0.15	5.587	5.587	5.555
0.20	4.262	4.262	4.223
0.25	3.484	3.484	3.442
0.30	2.978	2.979	2.960

**Table 3:** Comparison between the present results and those from Ref. [27].

	${\bar{\sigma }}_{z}\left(\times 10^{2}\right)$			${\bar{\sigma }}_{r}$		
*z/h*	Li et al. [27]	Present	Error%	Li et al. [27]	Present	Error%
−0.5	−1	−1	0.00	−0.1572	−0.1571	0.06
−0.4	−0.9696	−0.971	0.14	−0.1210	−0.1200	0.83
−0.3	−0.8907	−0.8907	0.00	−0.0878	−0.0877	0.11
−0.2	−0.7745	−0.7746	0.02	−0.0546	−0.0546	0.00
−0.1	−0.6326	−0.6325	0.01	−0.0234	−0.0233	0.43
0	−0.4827	−0.483	0.06	0.0063	0.0063	0.00
0.1	−0.3378	−0.3363	0.45	0.0354	0.0352	0.56
0.2	−0.2055	−0.2052	0.15	0.0621	0.0619	0.32
0.3	−0.0959	−0.0958	0.05	0.0868	0.0867	0.12
0.4	−0.0244	−0.0246	0.78	0.1124	0.1124	0.00
0.5	0	0	0.00	0.1330	0.1334	0.3

**Table 4:** Material properties for PZT-4 [30].

Property	PZT-4				
Elastic ($10^{9}\;N/m^{2}$)	$c_{11}^{0}=139$	$c_{12}^{0}=77.8$	$c_{13}^{0}=74.3$	$c_{33}^{0}=115$	$c_{44}^{0}=25.6$
Piezoelectric ($C/m^{2}$)	$e_{15}^{0}=12.7$	$e_{31}^{0}=-5.2$	$e_{33}^{0}=15.1$		
Dielectric ($10^{9}\;F/m$)	$\varepsilon_{11}^{0}=6.46$	$\varepsilon_{33}^{0}=15.62$			

## Data Availability

The original contributions presented in this study are included in the article. Further inquiries can be directed to the corresponding author.

## Appendix A

The expressions for the displacement function and the potential function are as follows:

$$\begin{array}{l} A_{0}=0,\;\;C_{0}=-g_{1}^{0}\left(0\right),\;\;G_{0}=g_{0}^{0}(0),\;\;C_{1}\left(z\right)=g_{1}^{0}\left(z\right),\;\;G_{1}\left(z\right)=-g_{0}^{0}(z),\\ F_{11}\left(z\right)=\kappa_{1}g_{6}\left(z\right)-h_{0}^{0}\left(z\right),\;\;F_{0}=-f_{40}\left(0\right)+\Phi_{1}f_{60}\left(0\right)-g_{0}^{1}\left(0\right)+\left(0+h/2\right)G_{0},\\ B_{11}\left(z\right)=\kappa_{2}g_{6}\left(z\right)+h_{1}^{0}\left(z\right),\;\;B_{0}=-f_{50}\left(0\right)+\Phi_{2}f_{60}\left(0\right)+g_{1}^{1}\left(0\right)+\left(0+h/2\right)C_{0},\\ D_{01}\left(z\right)=-\kappa_{3}f_{1}\left(z\right)-\kappa_{4}f_{2}\left(z\right),\;\;D_{11}=-c_{13}\left(z\right)\left[F\left(z\right)+B\left(z\right)\right]-q_{1},\\ D_{02}\left(z\right)=-\kappa_{3}\left[f_{10}\left(z\right)-\Phi_{1}f_{30}\left(z\right)\right]-\kappa_{4}\left[f_{20}\left(z\right)-\Phi_{2}f_{30}\left(z\right)\right],\\ D_{22}=-e_{13}\left(z\right)\left[F\left(z\right)\kappa_{3}+B\left(z\right)\kappa_{4}\right],\\ D_{0}=D_{00}\left(0\right),\;\;\Phi_{0}=\phi_{00}\left(0\right),\;\;\phi_{10}\left(z\right)=f_{0}^{0}\left(z\right),\;\;\phi_{20}\left(z\right)=-f_{1}^{0}\left(z\right),\\ \Phi_{2}=\frac{\kappa_{1}f_{20}\left(h/2\right)-\kappa_{2}f_{10}\left(h/2\right)+\kappa_{2}\Phi_{1}f_{30}\left(h/2\right)}{\kappa_{1}f_{30}\left(h/2\right)},\;\;\Phi_{1}=-fi_{0}^{0}\left(0\right), \end{array}$$

where

$$\begin{array}{l} g_{i}^{n}\left(z\right)={\int_{-h/2}^{z}\left(z-\xi \right)}^{n}\xi^{i}\frac{c_{13}\varepsilon_{33}+e_{33}e_{31}}{e_{33}^{2}+c_{33}\varepsilon_{33}}d\xi ,\;\;\;g_{6}(z)=\int_{-h/2}^{z}c_{44}d\xi ,\\ f_{i}^{n}\left(z\right)={\int_{-h/2}^{z}\left(z-\xi \right)}^{n}\xi^{i}\frac{c_{33}e_{31}-c_{13}e_{33}}{e_{33}^{2}+c_{33}\varepsilon_{33}}d\xi ,\;\;\left(i,n=0,1\right),\\ h_{i}^{n}\left(z\right)={\int_{-h/2}^{z}\left(z-\xi \right)}^{n}\xi^{i}\left(c_{11}-\frac{\varepsilon_{33}c_{13}^{2}+2c_{13}e_{33}e_{31}-c_{33}e_{31}^{2}}{e_{33}^{2}+c_{33}\varepsilon_{33}}\right)d\xi ,\;\;\left(n,i=0,1\right),\\ f_{40}\left(z\right)=\int_{-h/2}^{z}\left[\frac{\kappa_{1}g_{6}\left(\xi \right)-h_{0}^{0}\left(\xi \right)-e_{15}\phi_{10}\left(\xi \right)}{c_{44}}\right]d\xi ,\;f_{50}\left(z\right)=\int_{-h/2}^{z}\left[\frac{\kappa_{2}g_{6}\left(\xi \right)+h_{1}^{0}\left(\xi \right)-e_{15}\phi_{20}\left(\xi \right)}{c_{44}}\right]d\xi ,\\ f_{60}\left(z\right)=\int_{-h/2}^{z}\frac{e_{15}}{c_{44}}d\xi ,\;\;f_{1}(z)=\int_{-h/2}^{z}\left[\kappa_{1}g_{6}\left(\xi \right)-h_{0}^{0}\left(\xi \right)\right]d\xi ,\\ f_{2}(z)=\int_{-h/2}^{z}\left[\kappa_{2}g_{6}\left(\xi \right)+h_{1}^{0}\left(\xi \right)\right]d\xi ,\;\;f_{30}\left(z\right)=\int_{-h/2}^{z}\left(\frac{e_{15}^{2}}{c_{44}}+\varepsilon_{11}\right)d\xi ,\\ f_{10}\left(z\right)=\int_{-h/2}^{z}\left\{\frac{e_{15}}{c_{44}}\left[\kappa_{1}g_{6}\left(\xi \right)-h_{0}^{0}\left(\xi \right)\right]-\phi_{10}\left(\xi \right)\left(\frac{{e_{15}}^{2}}{c_{44}}+\varepsilon_{11}\right)\right\}d\xi ,\\ f_{20}\left(z\right)=\int_{-h/2}^{z}\left\{\frac{e_{15}}{c_{44}}\left[\kappa_{2}g_{6}\left(\xi \right)+h_{1}^{0}\left(\xi \right)\right]-\phi_{20}\left(\xi \right)\left(\frac{{e_{15}}^{2}}{c_{44}}+\varepsilon_{11}\right)\right\}d\xi ,\\ D_{00}\left(0\right)=\int_{-h/2}^{0}\left\{\frac{\varepsilon_{33}\left[D_{01}\left(\xi \right)+D_{11}\right]+e_{33}\left[D_{02}\left(\xi \right)+D_{22}\right]}{e_{33}^{2}+c_{33}\varepsilon_{33}}\right\}d\xi ,\\ \phi_{00}\left(0\right)=\int_{-h/2}^{0}\left\{\frac{e_{33}\left[D_{01}\left(\xi \right)+D_{11}\right]-c_{33}\left[D_{02}\left(\xi \right)+D_{22}\right]}{e_{33}^{2}+c_{33}\varepsilon_{33}}\right\}d\xi . \end{array}$$
